# Disease-syndrome combination modeling: metabolomic strategy for the pathogenesis of chronic kidney disease

**DOI:** 10.1038/s41598-017-09311-0

**Published:** 2017-08-18

**Authors:** Shasha Li, Peng Xu, Ling Han, Wei Mao, Yiming Wang, Guoan Luo, Nizhi Yang

**Affiliations:** 1 Guangdong Provincial Hospital of Chinese Medicine, No. 111 Dade Road, Guangzhou Guangdong, 510120 China; 2 Department of Chemistry, Tsinghua University, No. 30 Shuangqing Road in Haidian Distric, Beijing, 100084 China

## Abstract

Conventional disease animal models have limitations on the conformity to the actual clinical situation. Disease-syndrome combination (DS) modeling may provide a more efficient strategy for biomedicine research. Disease model and DS model of renal fibrosis in chronic kidney disease were established by ligating the left ureter and by ligating unilateral ureteral combined with exhaustive swimming, respectively. Serum metabolomics was conducted to evaluate disease model and DS model by using ultra performance liquid chromatography coupled with quadrupole time-of-flight mass spectrometry. Potential endogenous biomarkers were identified by multivariate statistical analysis. There are no differences between two models regarding their clinical biochemistry and kidney histopathology, while metabolomics highlights their difference. It is found that abnormal sphingolipid metabolism is a common characteristic of both models, while arachidonic acid metabolism, linolenic acid metabolism and glycerophospholipid metabolism are highlighted in DS model. Metabolomics is a promising approach to evaluate experiment animal models. DS model are comparatively in more coincidence with clinical settings, and is superior to single disease model for the biomedicine research.

## Introduction

As an absolutely essential part to most of the modern medicine research, animal models were widely used to investigate disease or injury states especially in ways which would be inaccessible in patients. Most of the animal models serving in research at present are disease models, which may have an existing, inbred or induced disease similar to a human condition, and thus may meet most of the requirement of western medicine researches.

However, people come to realize the limitation of an animal model^1^, that the duplicated disease in animal is often different from the disease in human being. Human diseases, in most cases, are multifactorial and thus often accompanied by complications or a series of other symptoms. For instance, most patients of renal fibrosis are also characterized with weakness, fatigue, dizziness, deficient sweating, stuffy chest, short breath and blood stasis, which are, according to the theory of traditional Chinese medicine (TCM), typical observations of syndrome of qi-deficiency and blood-stasis (QDBS). The most common rat model of renal fibrosis is established by ligation of the left ureter^2^, and validated with blood biochemistry and kidney histopathology indicators, without too much attention on other symptoms that should be present. As a result, this kind of simple disease animal model cannot reflect the real condition of human being, which might be, as least in part, responsible to the possible inconsistency between animal experiments and clinical trials^3^.

In theory and practice, TCM believed that disease does not exist in isolation, often accompanied by some specific “syndrome” (more accurately, maybe should be transliterated as “Zheng”)^4^. Disease-Syndrome (DS) combination modeling^5^, taking both disease and syndrome into consideration, is expected to be an efficient solution deal with the limitation of disease model. Then, how to evaluate the validity of an established DS model? A disease model can be evaluated by determination on some addressed biochemical indicators, which is, nevertheless, may not suffice to a DS model. Syndrome is a holistic concept with global pathological or physiological abnormalities occurred in patients or model animal, and can hardly be described by limited biochemical index^6^. Metabolomics is a holistic analysis strategy widely applied in the field of biomedicine research and in clinical application, and may be used as a powerful tool for the evaluation of TCM and DS models^7, 8^.

Chronic kidney disease (CKD) is a serious disease that jeopardizes human health worldwide^9^. The common pathological alteration in progressive CKD was tubulointerstitial fibrosis^10^. Tubulointerstitial fibrosis was complex, involving various independent and overlapping cellular and molecular pathways^11^. It was reported that metabolomics has been used to study patients with CKD for the last several years^12–16^. Compared with clinical aplication, metabolomics has been applied widely to the study of different animal models of CKD such as 5/6 nephrectomized CKD, adenine-induced chronic renal failure and other drug-induced CKD^17–23^. However, most publications were just based on the studies on animal disease models^24^. Statistic study of clinical cases in our hospital found that renal fibrosis patients had mostly a syndrome of qi-deficiency and blood-stasis (QDBS), and this conclusion has also been supported by literature in year 2010^25^. Ultra-high performance liquid chromatography-quadrupole time-of-flight mass spectrometry (UPLC-QTOF/MS) with rapidly separation and high reproducibility is regarded as a suitable for untargeted metabolomics in disease biomarker discovery and drug research^26–29^. In this present study, a DS model was established in rats as a combination model of QDBS and renal fibrosis in CKD, and metabolomic approach was used to comparatively evaluate this model. It is expected to discover novel diagnostic biomarkers and to highlight pathophysiological mechanisms of renal fibrosis in CKD.

## Materials and Methods

### Animals and Treatment

Thirty-two specific pathogen-free Sprague Dawley (SD) rats (male, weight, 180–220 g) were provided by the Experimental Animal Center of Guangdong Medical (certificate number, 0079361). All experiments were performed in accordance with the internationally accepted standard guidelines for the use of animals. This study was conducted in accordance with the Chinese national legislation and local guidelines, and the care and handling of rats were also approved by the Ethical Committee of Guangdong Provincial Hospital of Chinese Medicine. The rats were housed under standard environmental conditions (23 ± 2 °C, 55% ± 5% humidity and 12 h/12 h light/dark cycle) and were allowed free access to water as well as standard laboratory rat food.

After 3 days of acclimatization, the rats were randomly divided into three groups of control group (n = 10), disease model group (n = 10) and DS model group (n = 12), respectively. Rats in disease and DS model group underwent ligation of the left ureter to establish a model of renal fibrosis, and rats in DS group were further forced to swim exhaustively every day to establish a DS model; they swam in a pool of water with a depth of 60 cm at room temperature and were picked up when 50% of the rats were exhaustively submerged in water for 10 s. This experiment lasted for 28 days.

### Sample Preparation

Three groups of rats were sacrificed after being anesthetized by intraperitoneal injection of pentobarbital (50 mg/kg of body weight) (GBCBIO Technologies, Guangzhou, China) and their blood and kidney were collected. Blood samples were drawn into tubes, allowed to stand for 30 min, and were centrifuged to obtain serum. Serum samples were snap frozen in liquid nitrogen for 5 min and then stored in a refrigerator at −80 °C. Kidney tissues were fixed with 10% formalin (Sigma, America) in PBS for 12 h after dehydration, cleared in xylene (Damao chemical reagent company, Tianjin, China), penetrated in wax and embedded in paraffin (Leica, Germany).

Thawed serum (400 μL) was mixed with methanol (1200 μL), then swirled for 2 min and centrifuged at 13,000 rpm for 15 min at 4 °C. The clear supernatant was transferred and evaporated to dryness by N_2_, followed by dilution with 300 µL of methanol-H_2_O (80:20, v/v) (Merck, Germany) and centrifugation at 13,000 rpm for 15 min at 4 °C. The supernatant was transferred to a new EP tube for UPLC-QTOF/MS analysis.

### Chromatographic Condition

Chromatography was performed on a Waters Acquity UPLC BEH C_18_ column (2.1 mm × 100 mm, 1.7 μm) at 30 °C. The mobile phase consisted of acetonitrile (Merck, Germany) as solvent A and 0.1% formic acid (Merck, Germany) in water as solvent B. The gradient programs was as follows: 0–5 min, 2–50% A; 5–7 min, 50–60% A; 7–14 min, 60–65% A; 14–18 min, 65–80% A; 18–24 min, 80–90% A; 24–26 min, 90–95% A; 26–28 min, 95–2% A; and 28–30 min, 2% A. The injection volume of the test sample was 5 µL and each sample was injected three times. Each wash cycle consisted of 200 µL of strong wash solvent (80% CH_3_CN-H_2_O, 8:2, v/v) and 600 µL of weak wash solvent (10% CH_3_CN-H_2_O, 1:9, v/v).

### Mass spectrometry condition

MS was performed on a Waters Q-TOF Premier with an electrospray ionization system (Waters MS technologies, Manchester, UK). A preliminary experiment was conducted to optimize the experimental conditions. To achieve the desired detection results, the flow rate and column temperature for chromatography, as well as capillary voltage, flow and temperature of the desolvation gas for the mass spectrometry detector were optimized carefully. As a result, the optimal parameters were fixed as followed. The capillary voltages were set at 3.0 and 2.5 kV, and the sample cone voltages were 30 and 45 V for the positive and negative ion modes, respectively. The desolvation gas flow was set to 600 L/h at a desolvation temperature of 350 °C, the cone gas set to 50 L/h and the source temperature was 110 °C. The Q-TOF Premier acquisition rate was 0.1 s with a 0.02 s inter-scan delay. The instrument was operated with the first resolving quadrupole in a wide-pass mode (50–1500 Da). Argon was used as the collision gas. ESI-MS spectra were acquired in both ionization modes of positive and negative with full-scan detection.

The precision and stability of the UPLC-MS method was determined by repeated analysis of six injections of the same QC samples, and the repeatability of sample preparation was accessed by preparing six parallel samples using the same protocol. All assays were performed using a lock spray to ensure accuracy and reproducibility, with Leucine enkephalin amide acetate as the lock mass at 200  рg/μL concentration and 20 μL/min flow rate.

### Data processing

Data pre-processing was performed with MarkerLynx applications manager version 4.1 (Waters, MA, USA). The parameters included retention time from 1 min to 26 min, mass range from 50 Da to 1500 Da and mass tolerance was 0.01 Da. The minimum intensity was set at 10% of base peak intensity, the maximum mass per retention time was set at 6 and the retention time tolerance was 0.05 min. MarkerLynx incorporates an Apex-Track-peak detection package and the data were combined into a single matrix by aligning peaks with the exact mass/retention time pair together. The ion intensities for each peak were normalized to a constant integrated intensity to partially compensate for the concentration bias of each sample.

The processed data was then exported and processed by principle component analysis (PCA) and partial least square-discriminant analysis (PLS-DA) in the software package SIMCA-P 11.5 version (Umetrics AB, Umeå, Sweden). PCA was used first to determine the general interrelation between the three groups, and PLS-DA was subsequently performed to maximize the difference in metabolic profiling. The value of Q^2^ and R^2^ were used to estimate the accuracy of the model and a typical 7-round cross-validation was used to validate the model against over fitting.

Statistical analyses were performed using SPSS software (Version 18.0, USA). Assumptions of normality and homogeneity of variance were first checked. Data were presented as the mean ± standard deviation for continuous variables with a normal distribution, as counts and percentages for categorical variables. The independent samples *t*-test or one-way ANOVA were used to analyze the differences among groups for continuous measures. Differences with *P* values less than 0.05 were considered to have statistical differences, and *P* values less than 0.01 were considered to have significant differences. All probability values were two-sided.

### Data availability

All data generated during this study are included in this published article and the raw data are available from the corresponding author on reasonable request.

## Results

### Change of weight and swimming time of rats

**Figure 1:**
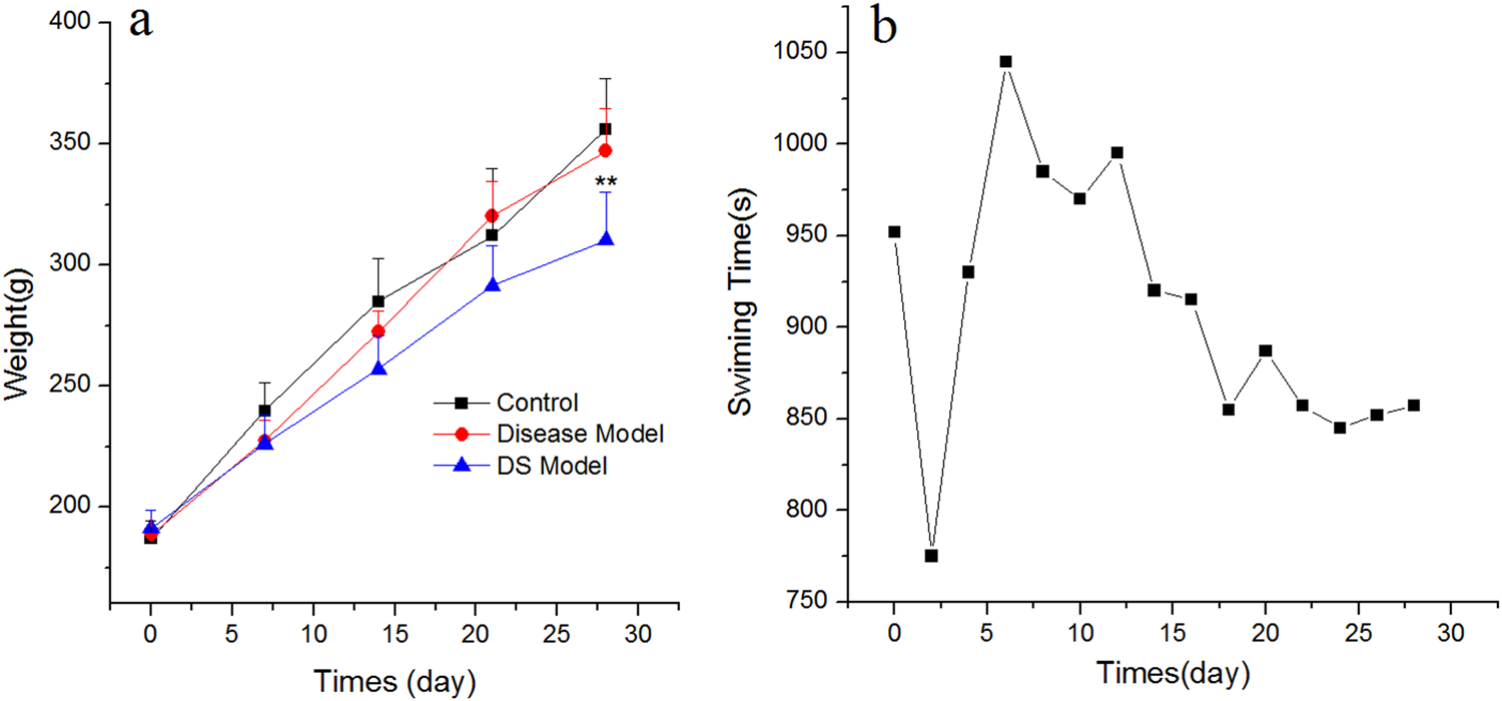
The change of weight in all three groups and swimming time in DS model group. The weight of all rats was measured weekly and the swimming time of the rats for the DS model group was measured every 2 days from the start to the end of the experiment. Rats in DS model group were weighed before exhaustive swimming. Data were reported as the mean ± standard deviation in each group. *P < 0.05, compared with the control group. The swimming time was calculated from the point when rats were placed in water to the point when 50% of the rats were exhaustively submerged in water for 10 s.

The weight of all rats was recorded every week, and the swimming time of the rats for the DS model group was measured every 2 days. Figure 1a shows that the weight of all rats increased with time. Starting from the second week, the weight growth rate of the control group and disease model group was faster than that of DS model group, and this phenomenon persisted until the end of the experiment. The Dunnett’s test showed that the weights of the rats in each group were equal at the beginning of the experiment; however, the final weights of the DS model group were significantly lower than that of the control group and the disease group (P = 2.92 × 10^−6^ and 1.05 × 10^−3^, respectively). No significant difference was observed between the control and disease model group. Figure 1b shows the exhaustive swimming time of the DS model group during the adaptive swimming period. After stress reduction in the second day, the swimming time of rats shortened gradually in followed two weeks. Then, the time remained relatively stable until the end of the experiment.

### Serum biochemistry

Thirty-two rats were included in this study and the results of serum biochemistry are listed in Table 1. Compared with control rats, rats of both model groups show significant changes in levels of cholesterol, lactate dehydrogenase, creatine kinase, blood urea nitrogen and creatinine. However, excepting triglyceride, these biochemical parameters in rats showed no statistical differences between disease group and DS group.

**Table 1:** Comparison of serum biochemistry analysis among groups^*a, b*^.

	Control group	Disease model group	DS model group
No. of subjects	10	10	12
Cholesterol, mmol/L	1.18 ± 0.20	2.56 ± 0.30**	2.66 ± 0.29**
Triglyceride, mmol/L	0.37 ± 0.10	0.57 ± 0.08**	0.37 ± 0.12^ΔΔ^
Lactate dehydrogenase, U/L	429.20 ± 89.17	1131.11 ± 122.93**	1111.86 ± 91.61**
Creatine kinase, U/L	225.80 ± 38.88	416.92 ± 95.67**	443.38 ± 80.69**
Blood urea nitrogen, mmol/L	7.40 ± 1.55	9.90 ± 1.06**	9.32 ± 1.13*
Creatinine, μmol/L	14.75 ± 2.75	54.60 ± 8.40**	55.29 ± 13.46**

^a^The serum levels of biochemical indices of rats are presented as the mean ± standard deviation. ^*b*^The serum concentrations of biochemical indices were statistically tested by Dunnett’s *t*-test when the variance in different groups was homogeneous and by the Games-Howell test when the variance was non-homogeneous. *P < 0.05, **P < 0.01 compared with the control group; ^Δ^ *P* < 0.05, ^ΔΔ^ *P* < 0.01 compared with the disease model group.

### Immunohistochemical staining

**Figure 2:**
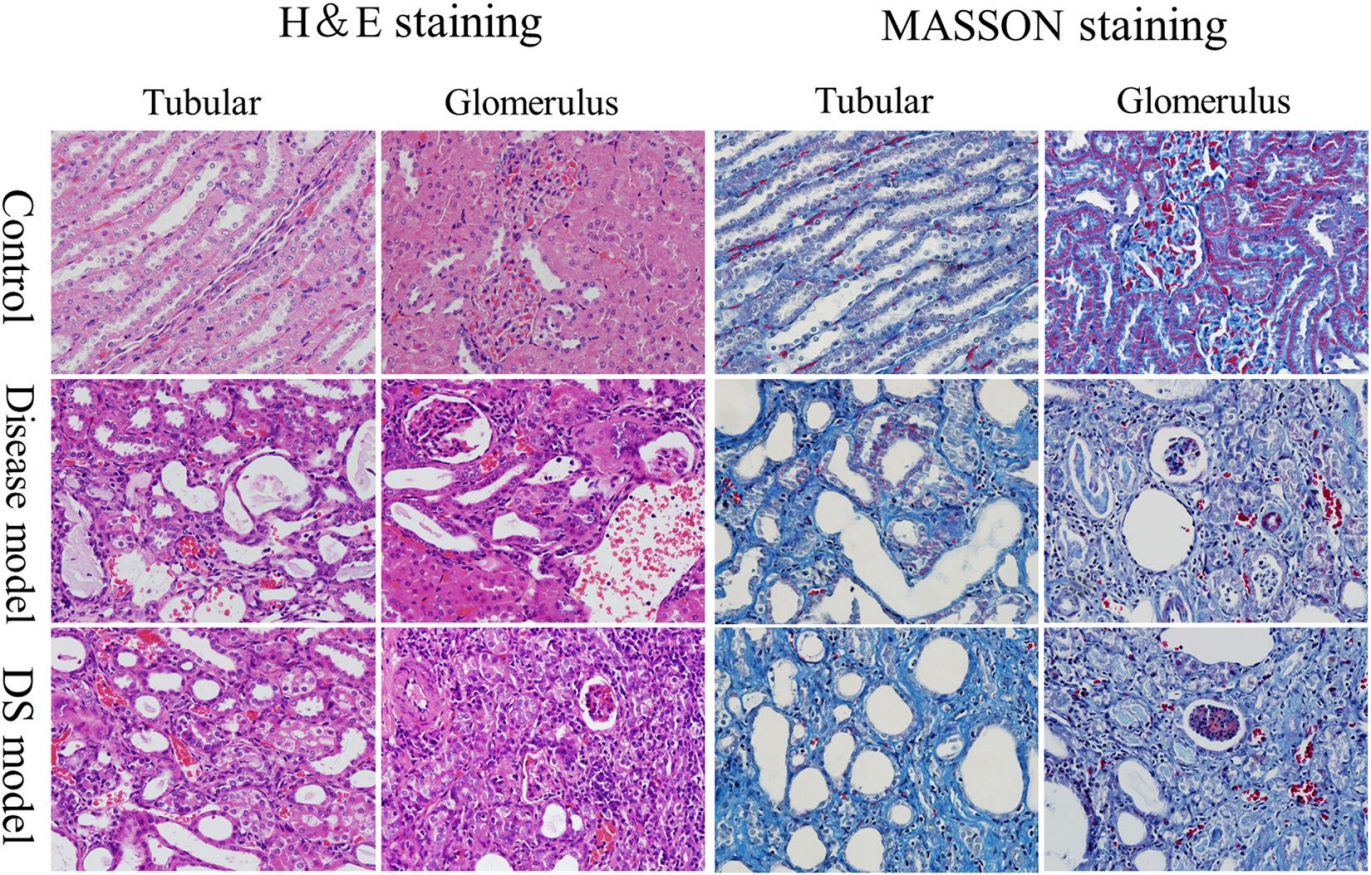
H& E and Masson staining on kidney tissue of rats in control, disease and DS groups. H& E staining: rats in disease and DS groups were observed with renal cortex and medulla atrophied, glomerular sclerosis and atrophy, renal tubular expansion, structure diffusion, visible hyalinization, protein tube and a large number of cavity; Glomerular and capillary congestion and clear inflammatory cell infiltration was also observed. Masson staining: glomerular basement membrane and tubule interstitial fibers proliferated significantly in disease and DS groups of rats.

Paraffin embedded kidney tissue were cut into 4 μm sections. Hematoxylin & Eosin (H&E) and Masson staining were performed on sections for observation of kidney pathohistological changes. As illustrated in Fig. 2, compared with control group, those in disease and DS groups were observed with renal cortex and medulla atrophied, and microscopic examination showed the development of glomerular sclerosis and atrophy by H&E staining, together with renal tubular expansion, structure diffusion, visible hyalinization, protein tube and a large number of cavity; in addition, glomerular and capillary congestion and clear inflammatory cell infiltration was also observed in disease and DS groups of rats. Masson staining showed dramatically tubulointerstitial fibrosis. Immunohistochemical staining indicates both disease and DS models successfully established renal fibrosis models, while it cannot show any significant variations between them.

### Metabolic Fingerprinting

**Figure 3:**
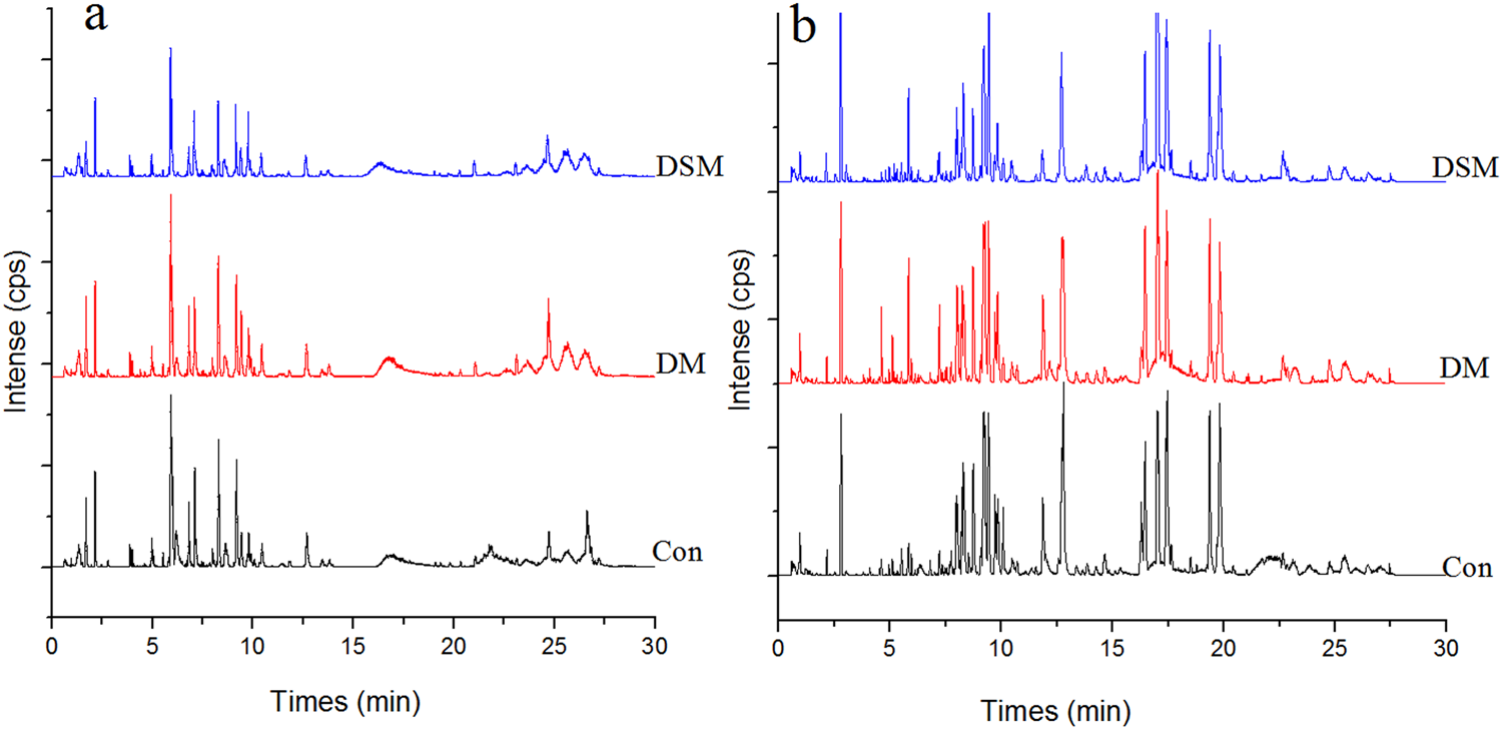
Representative base peak intensity chromatogram of rat serum obtained in the ESI positive (**a**) and negative ion modes (**b**) based on UPLC-QTOF/MS. Con, control group; DM, disease model group; DSM, DS model group.

Based on the optimal conditions, the UPLC-MS base peak intensity chromatograms obtained in the ESI positive and negative ion modes are shown in Fig. 3. In this study, abundant chromatographic peak information was observed both in the positive and negative ion modes. To acquire the maximum amount of detectable metabolites, the detection data of two ionization patterns were all considered in further study. Quasi-molecular ion [M + H]^+^ was the most usually form with a higher signal/noise in the positive ion mode and [M − H]^−^ or [M + HCOO]^−^ in the negative ion mode.

The relative standard deviations of the peak retention time and area value in methodological investigation were all less than 5.0%, indicating that the precision and repeatability of the proposed method were satisfactory for metabolomic analysis.

### PCA and PLS-DA

**Figure 4:**
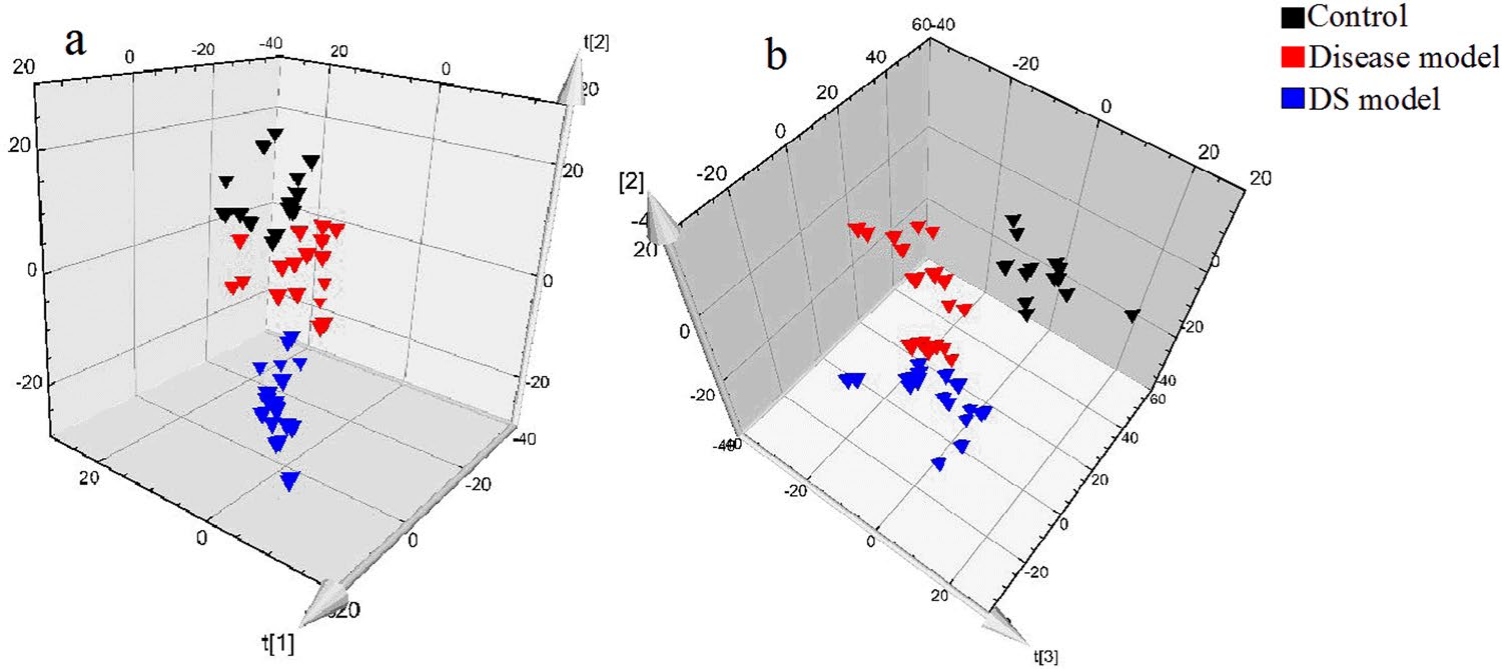
PCA 3D Score plots of the serum profiles in positive ion mode (**a**) and negative ion mode (**b**).

In this study, PCA was used first to determine the general interrelation between the three groups. For a clearer display of clustering, PCA 3D score plots were provided in this study. As shown in Fig. 4, the model groups and control group were separated clearly, indicating that the rats of model groups have significantly altered endogenous metabolism both in the positive and negative ion modes. Furthermore, the assembly of DS model group was far from control group compared to the disease model group both in positive and negative ion modes, indicating a more obvious change in metabolic state of DS model group. This indicates that metabolic fingerprints could reflect the alternation between disease and normal rats. In addition, metabolic fingerprints could exhibit the difference between disease model group and DS model group that cannot be observed in detection of serum biochemistry and immunohistochemical staining.

**Figure 5:**
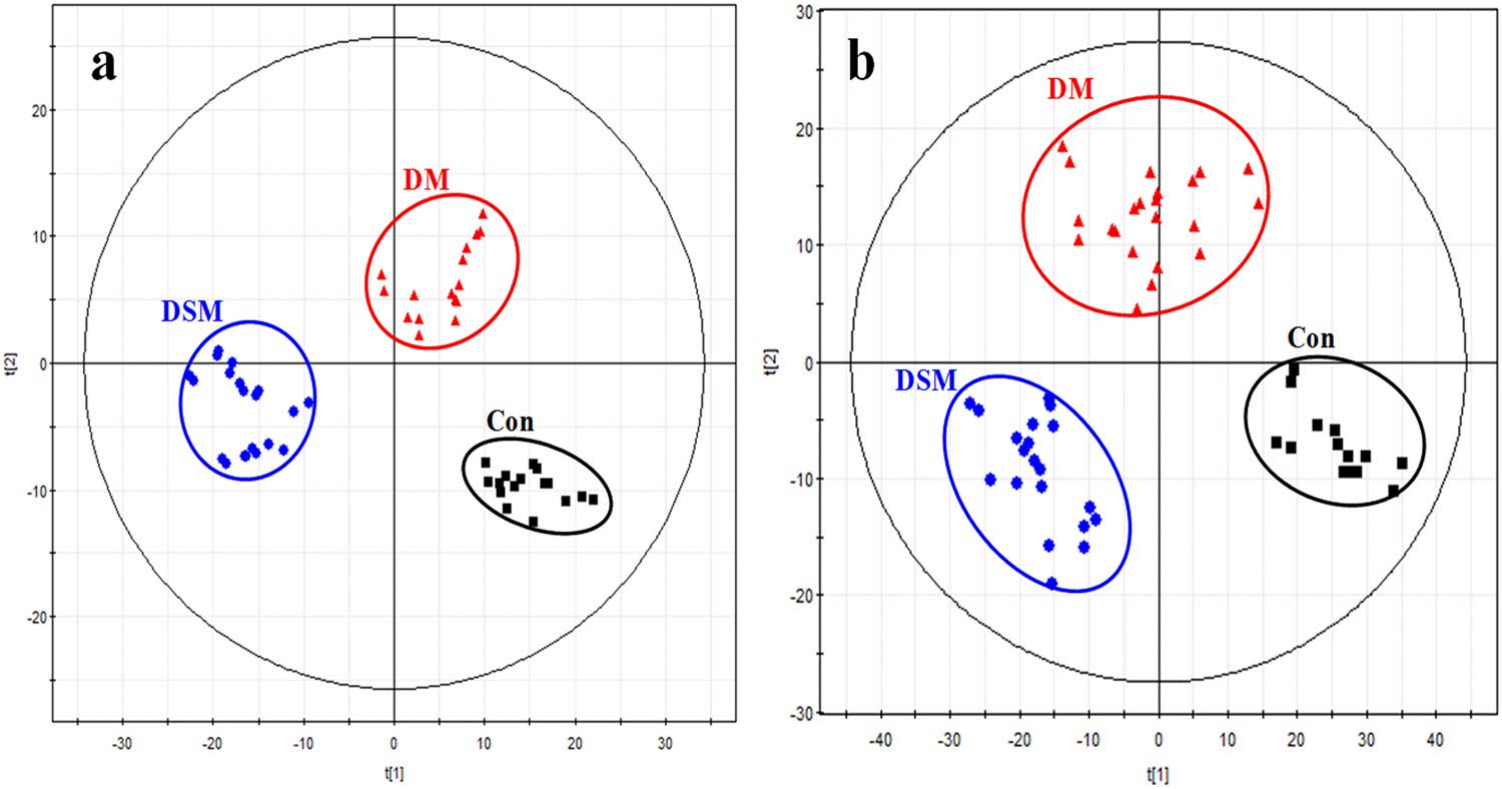
PLS-DA score plots of control group and model groups. (**a**) Comparison of groups in positive ion mode (R^2^X(cum) = 0.349, R^2^Y(cum) = 0.990, Q^2^Y(cum) = 0.944); (**b**) Comparison of groups in negative ion mode (R^2^X(cum) = 0.279, R^2^Y(cum) = 0.989, Q^2^Y(cum) = 0.919). Con, control group; DM, disease model group; DSM, DS model group.

Subsequently, PLS-DA was performed to maximize the difference in metabolic profiling (Fig. 5). The R^2^Y and Q^2^Y values calculated from the results in SIMCA-P package were 0.990 and 0.944 in the positive ion mode, meaning 99% of data fit the model and 94.4% of data could be predicted by this model. Besides, the R^2^Y and Q^2^Y values in the negative ion mode were 0.989 and 0.919 respectively. Both the Q^2^Y and R^2^Y close to 1 indicate an excellent model which is good to fitness and prediction. In order to maximize the difference, Data of control model and disease model or DS model groups were performed PLS-DA, respectively.

### Biomarkers

**Figure 6:**
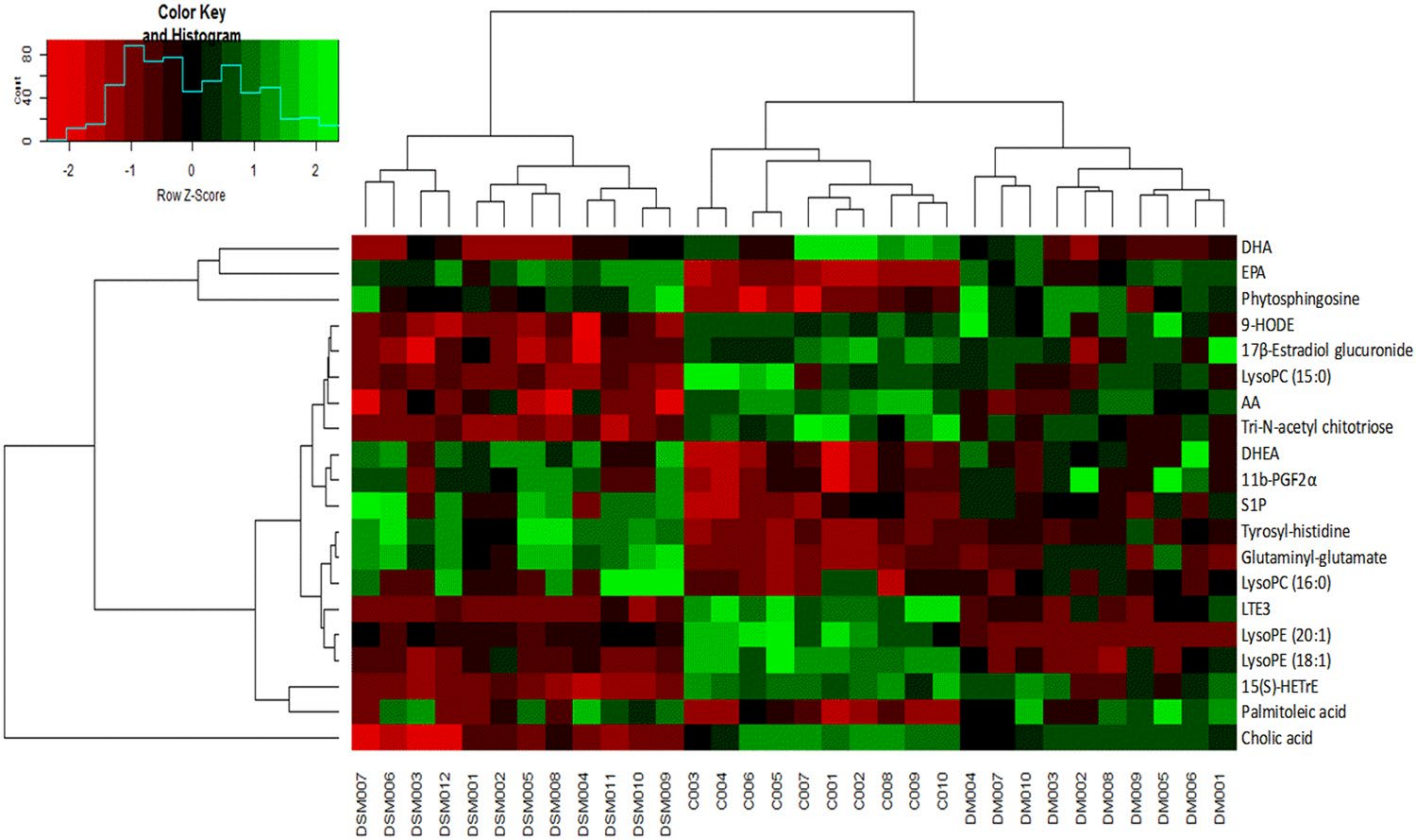
Heatmap of 20 potential biomarkers between control and model groups. The color of each section is proportional to the significance of change of metabolites (red, upregulated; green, downregulated)as the numbers listed in Table 2. Rows: metabolites; Columns: samples (C: control group; DM: disease model group; DSM: DS model group).

After PLS-DA processing, metabolites that significantly contributed to the clustering and discrimination were identified when the variable importance in the projection values was more than 1 and p-value of *t*-test between groups was less than 0.05. These endogenous metabolites were selected for further structural identification. In next process, the calculated mass, mass deviation (mDa and ppm), double bond equivalent, formula, and isotopic pattern of the selected ion (*i*-fit value) were used to evaluate the accuracy of possible formulas^30, 31^. A smaller mass deviation and a lower *i*-fit value indicate a more accurate elemental composition. The structural information of the metabolites selected was subsequently obtained by searching the freely accessible databases HMDB (http://www.hmdb.ca) and KEGG (http://www.genome.jp). Finally, twenty significantly differential metabolites were identified (Table 2). Moreover, the relative levels of biomarkers were also analyzed and were shown in Table 2, and the heatmap of these 20 potential biomarkers is shown in Fig. 6.

**Table 2:** Identification of significantly different potential endogenous metabolites in the sera of individuals in the control and model groups^*a*^.

No.	Identified potential biomarker	Related metabolism	Concentration			F^*c*^	Sig.^*c*^	*p* value^*d*^	
			Control	Disease model^*b*^	DS model^*b*^			Con. VS DM	Con. VS DSM
**1**	Leukotriene E3	Arachidonic acid metabolism	34.54 ± 5.67	14.81 ± 6.44↓	7.94 ± 2.27↓	81.62	1.23 × 10^−12^	2.52 × 10^−8^	2.33 × 10^−8^
**2**	15(S)-Hydroxyeicosatrienoic acid	γ-Linolenic acid metabolism	124.22 ± 14.73	101.64 ± 29.31↓	44.29 ± 7.87↓	53.00	2.07 × 10^−10^	2.27 × 10^−2^	2.37 × 10^−8^
**3**	LysoPE (20:1)	Glycerophospholipid metabolism	14.93 ± 4.49	1.78 ± 0.82↓	5.80 ± 1.40↓	63.35	2.61 × 10^−11^	2.33 × 10^−8^	4.16 × 10^−8^
**4**	Tyrosyl-histidine	Tyrosine metabolism	16.88 ± 1.46	21.01 ± 2.26↑	29.40 ± 3.92↑	56.02	1.10 × 10^−10^	5.56 × 10^−3^	2.34 × 10^−8^
**5**	Glutaminyl-glutamate	Glutamate metabolism	17.29 ± 1.19	21.12 ± 3.58↑	27.65 ± 3.60↑	32.61	3.79 × 10^−8^	1.67 × 10^−2^	4.25 × 10^−8^
**6**	Sphingosine 1-phosphate	Sphingolipid metabolism	34.68 ± 6.52	43.28 ± 5.38↑	52.80 ± 12.55↑	11.01	2.78 × 10^−4^	1.45 × 10^−2^	1.22 × 10^−4^
**7**	Docosahexaenoic acid	α-Linolenic acid metabolism	215.29 ± 21.48	288.42 ± 37.48↑	283.26 ± 32.33↑	17.50	1.04 × 10^−5^	2.59 × 10^−5^	3.96 × 10^−5^
**8**	8,11,14-eicosatrienoyl ethanolamide	α-Linolenic acid metabolism	26.96 ± 6.69	41.47 ± 9.72↑	46.69 ± 9.00↑	15.01	3.35 × 10^−5^	1.42 × 10^−3^	1.84 × 10^−5^
**9**	11b-PGF2α	Arachidonic acid metabolism	26.17 ± 7.08	43.35 ± 11.74↑	41.89 ± 8.64↑	10.71	3.29 × 10^−4^	5.52 × 10^−4^	9.03 × 10^−4^
**10**	Palmitoleic acid	Fatty Acid Biosynthesis	57.01 ± 11.35	94.07 ± 18.91↑	85.33 ± 23.24↑	10.66	3.38 × 10^−4^	2.65 × 10^−4^	2.87 × 10^−3^
**11**	Cholic acid	Bile Acid Biosynthesis	39.07 ± 12.93	144.96 ± 31.70↑	166.96 ± 26.46↑	78.00	2.14 × 10^−12^	2.38 × 10^−8^	2.33 × 10^−8^
**12**	Arachidonic acid	Arachidonic acid metabolism	35.62 ± 3.81	30.06 ± 8.90↓	16.72 ± 5.57↓	25.73	3.74 × 10^−7^	1.09 × 10^−1^	2.96 × 10^−7^
**13**	Eicosapentaenoic acid	Α-Linolenic acid metabolism	245.94 ± 56.43	153.10 ± 39.18↓	122.45 ± 39.35↓	21.35	1.99 × 10^−6^	1.58 × 10^−4^	1.18 × 10^−6^
**14**	9-HODE	Linoleic acid metabolism	35.18 ± 1.40	35.76 ± 7.15	21.41 ± 4.15↓	32.27	4.22 × 10^−8^	9.47 × 10^−1^	4.88 × 10^−7^
**15**	Phytosphingosine	Sphingolipid metabolism	461.18 ± 55.03	403.14 ± 35.19↓	180.93 ± 66.80↓	81.25	1.30 × 10^−12^	4.57 × 10^−1^	2.33 × 10^−8^
**16**	LysoPC (16:0)	Glycerophospholipid metabolism	13.97 ± 5.31	16.45 ± 2.99↑	22.59 ± 9.59↑	4.73	1.67 × 10^−2^	6.29 × 10^−1^	1.16 × 10^−2^
**17**	LysoPE (18:1)	Glycerophospholipid metabolism	10.57 ± 1.24	4.47 ± 1.97↓	4.01 ± 1.27↓	60.62	4.38 × 10^−11^	2.47 × 10^−8^	2.34 × 10^−8^
**18**	LysoPC (15:0)	Glycerophospholipid metabolism	42.71 ± 5.02	29.30 ± 8.41↓	17.36 ± 10.69↓	23.96	7.20 × 10^−7^	2.88 × 10^−3^	2.84 × 10^−7^
**19**	Tri-N-acetyl chitotriose	Amino sugar metabolism	52.54 ± 10.38	38.94 ± 7.02↓	23.61 ± 4.99↓	39.54	5.20 × 10^−9^	7.98 × 10^−4^	2.52 × 10^−8^
**20**	17β-Estradiol glucuronide	Steroid hormone biosynthesis	41.38 ± 11.24	30.78 ± 6.30↓	17.92 ± 3.17↓	27.42	2.06 × 10^−7^	6.56 × 10^−3^	1.04 × 10^−7^

^*a*^Metabolites were confirmed by *t*_R_ and *m*/*z* with authentic chemicals. ^*b*^Compared with control group. “↑” indicates a higher level of metabolites, whereas “↓” represents a lower level of metabolites. All data are represented the intensity values of metabolites. ^*c*^The value of F and Sig. was obtained from a one-way ANOVA. The F value is the ratio of the variance between the groups and the variance in the group. F value greater than 1 and Sig. less than 0.05 means the differences between groups were statistically significant. ^*d*^ *p* values were calculated from the Dunnett test in multivariate statistical analysis.

**Figure 7:**
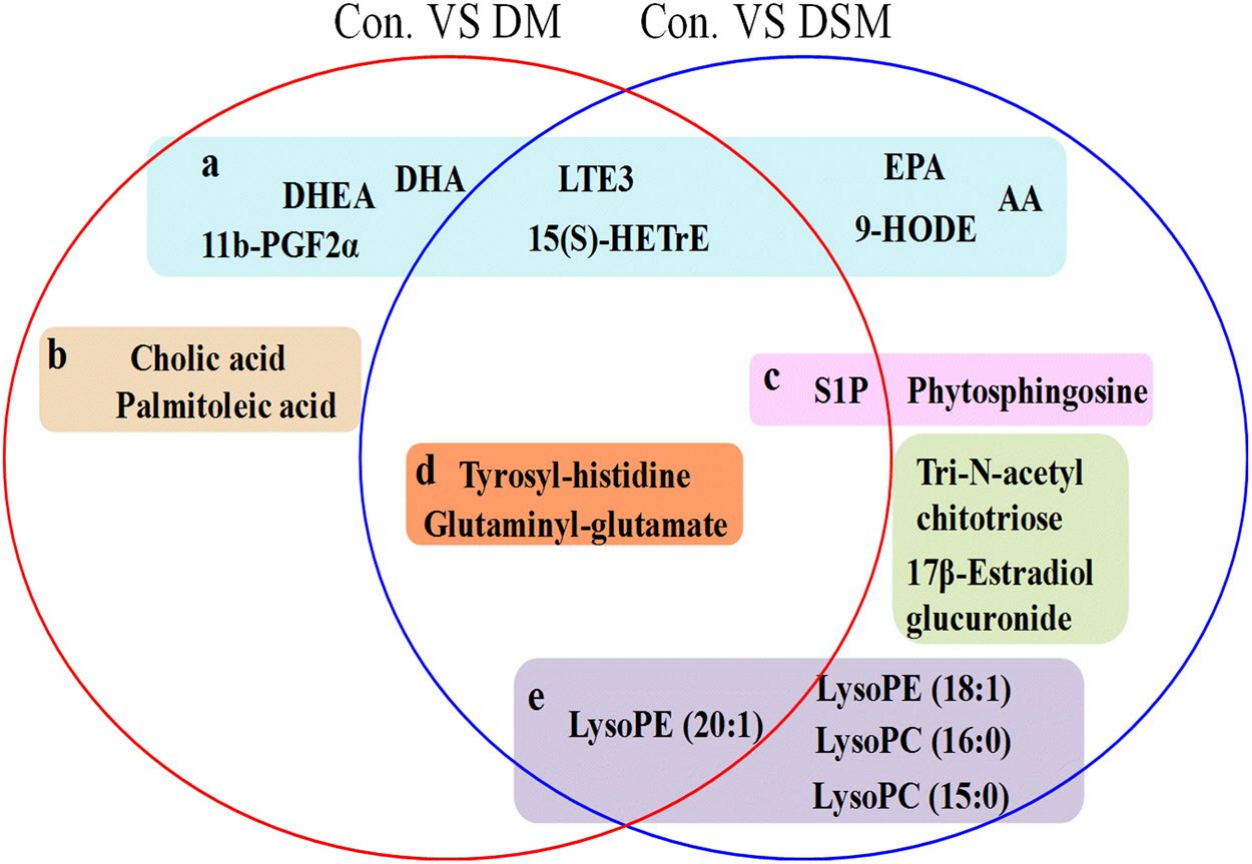
Identification of potential metabolites related to disease model rats and DS model rats. The color represent different classes of biomarkers: (**a**) arachidonic acid metabolism, linoleic acid metabolism; (**b**) fatty acid biosynthesis; (**c**) sphingolipid metabolism; (**d**) amino acid metabolism; (**e**) glycerophospholipid metabolism. DHA: docosahexaenoic acid; LTE3: leukotriene E3; 15S-HETrE: 15(S)-hydroxyeicosatrienoic acid; DHEA: 8,11,14-eicosatrienoyl ethanolamide; EPA: eicosapentaenoic acid; AA: arachidonic acid; S1P: Sphingosine 1-phosphate.

Eleven and fifteen potential biomarkers were identified in disease model and DS model, respectively. Leukotriene E3(LTE3), 15(S)-Hydroxyeicosatrienoic acid (15(S)-HETrE), LysoPE (20:1), Tyrosyl-histidine, Glutaminyl-glutamate and Sphingosine 1-phosphate(S1P) were observed as potential biomarkers in both models and these biomarkers show the same variation trends. No. 7–11 and No. 12–20 metabolites were particular biomarkers obtained by PLS-DA analysis in disease model and DS model, respectively (Table 2 and Fig. 7). The relative level of all twenty potential biomarkers was listed in Table 2 and a one-way ANOVA was conducted to evaluate the significance of difference between groups. The *p*-value in Table 2 showed that the relative level of five among No.12–20 had significant difference in disease model compared to control group, and No.7–11 metabolites had significant difference in DS model. This result illustrated again that the DS model could exhibit the abnormal endogenous metabolic status comprehensively.

**Figure 8:**
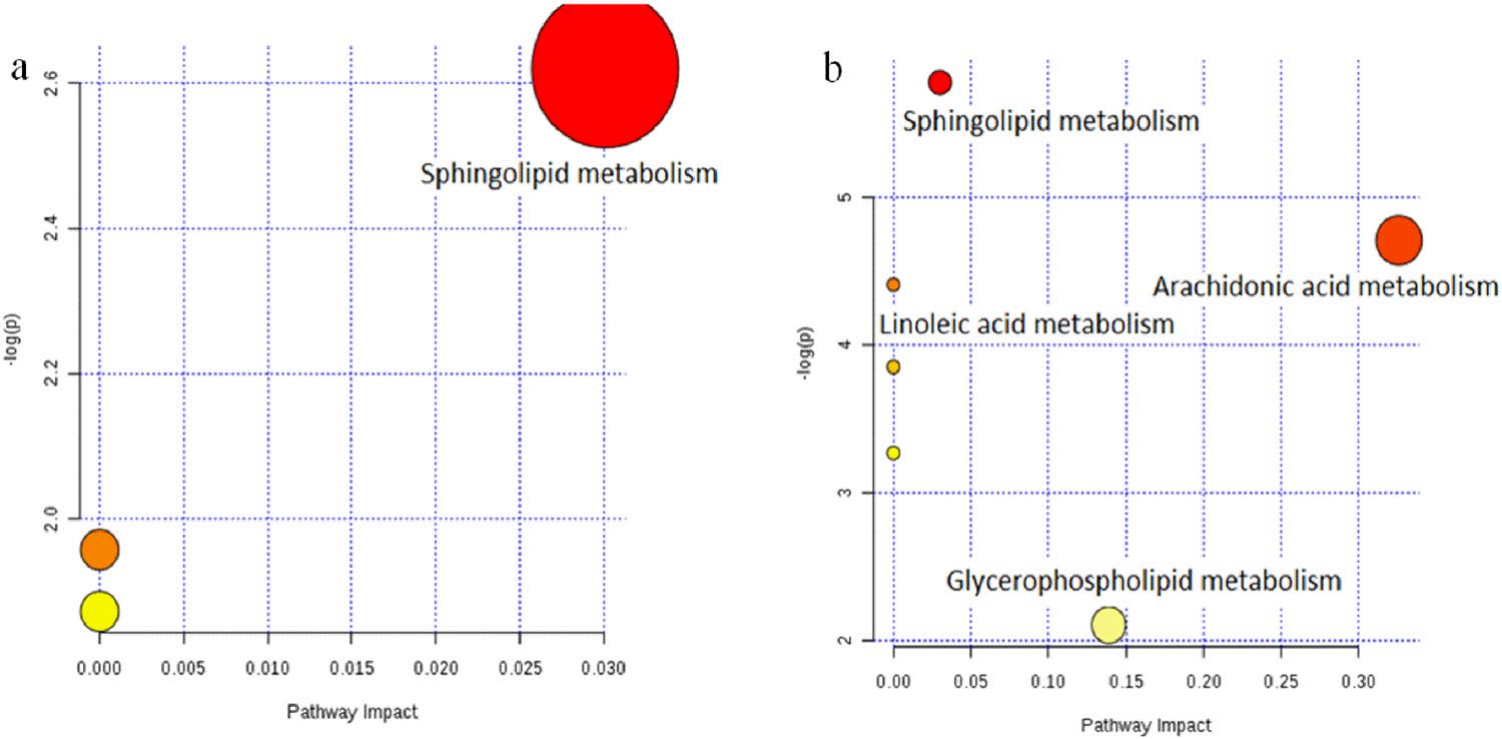
Pathway analysis on biomarkers of disease model (**a**) and DS model (**b**). All matched pathways according to p-values from pathway enrichment analysis and pathway impact values from pathway topology analysis, using pathway library of Rattus norvegicus (rat).

**Figure 9:**
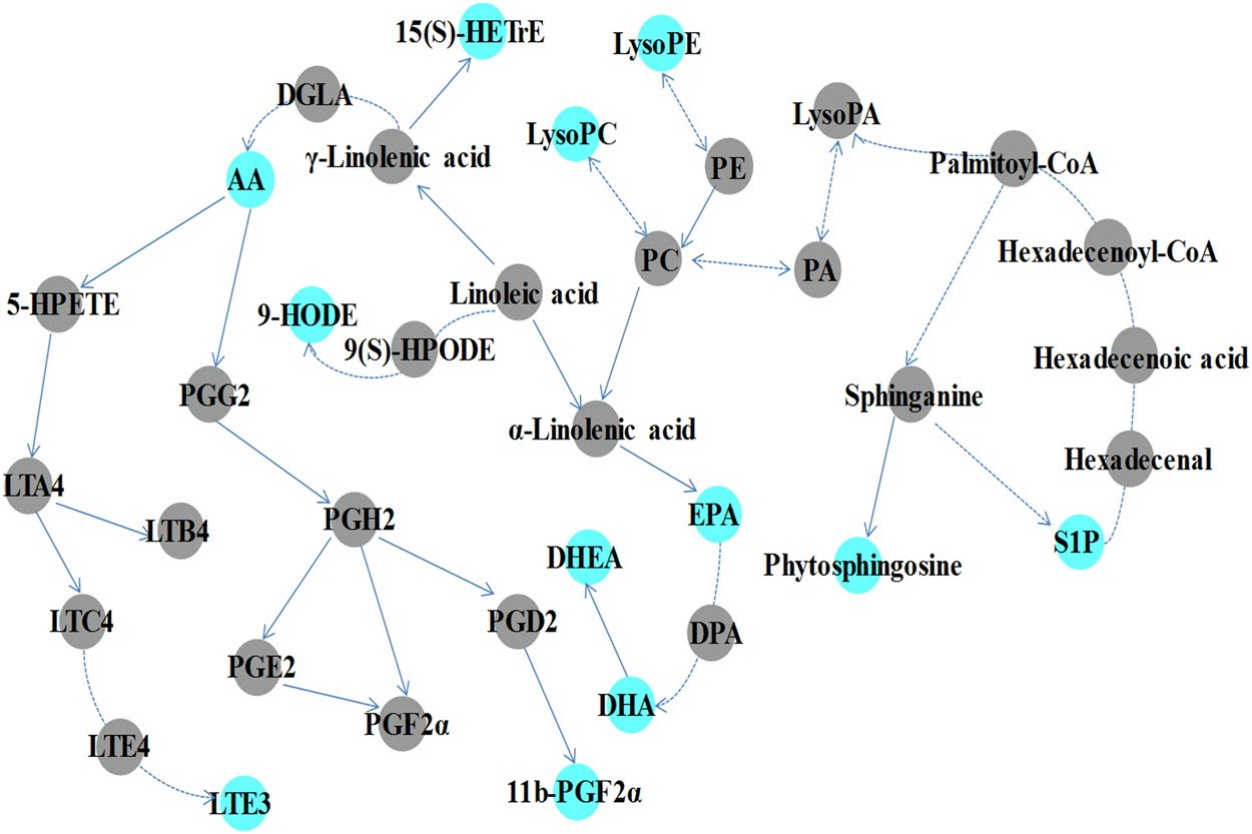
Biosynthetic pathways for parts of potential biomarkers related to arachidonic acid metabolism, linoleic acid metabolism, glycerophospholipid metabolism and sphingolipid metabolism. The colored dots showed metabolites identified in the current study. DGLA: dihomo-γ-linolenic acid; 15S-HETrE: 15(S)-hydroxyeicosatrienoic acid; AA: arachidonic acid; LTE3: leukotriene E3; EPA: eicosapentaenoic acid; DHA: docosahexaenoic acid; DHEA: 8, 11, 14-eicosatrienoyl ethanolamide; S1P: Sphingosine 1-phosphate.

Metabolic pathway analysis revealed key pathways involved in disease model and DS model^32, 33^. According to Fig. 8, abnormal sphingolipid metabolism is a common characteristic of both models, while there are some other important pathways of arachidonic acid (AA) metabolism, linoleic acid metabolism and glycerophospholipid metabolism are involved highlight in DS model. The biosynthetic pathways for parts of potential biomarkers related to arachidonic acid metabolism, linoleic acid metabolism, glycerophospholipid metabolism and sphingolipid metabolism were shown in Fig. 9.

## Discussion

In clinic, serum creatinine, blood urea nitrogen, cholesterol and triglyceride levels are the main biochemical biomarkers that are preferred to detect patients with nephropathy. Among these biomarkers, serum creatinine and blood urea nitrogen levels are most reliable for the detection of renal function, whereas cholesterol and triglyceride levels are related to lipid metabolism^34, 35^. In addition, creatine kinase and lactate dehydrogenase levels were used to access the physical condition of rats in the present experiment. This study applied two approaches to establish models for the same disease, including a common disease model and a disease model combined syndrome under the guidance of the TCM theory. Table 1 indicates that the two types of modeling methods can induce injury of the kidneys, which presented as damaged renal function, reduced physical condition and disordered the lipid metabolism. Except triglyceride, all biochemical indices in two models show significant variance compared with control rats, which indicate the validity of both models. The difference in triglyceride levels observed between the disease and DS model groups may be due to daily swimming, which requires a great amount of energy.

Disease model and DS model show no difference in biochemical biomarkers except triglyceride and suggests common approach is not sufficient to evaluate DS modeling. Metabolomics focuses on the quantitative and qualitative analysis of low-molecular-weight metabolites and is an efficient tool for globally understanding of diseases pathogeneses. Disease model and DS model have almost the same biochemical parameters, while metabolomics may highlight their difference. Besides 6 biomarkers in common, five metabolites were discovered only in disease model and control groups; while investigation on DS model and control groups identified nine particular biomarkers. MataboAnalyst 3.0 is efficient tool for metabolites related metabolic pathway analysis, and it indicates that abnormal sphingolipid metabolism is a common characteristic of both model, while AA metabolism, linoleic acid metabolism and glycerophospholipid metabolism are highlighted in DS model.

It has been reported that CKD was closely related to the abnormal sphingolipid metabolism^36, 37^, and kidney injury may be caused by excessive accumulation of sphingolipids^38–40^. In this study, two important metabolites phytosphingosine and sphingosine 1-phosphate (S1P) involved in sphingolipid pathway could be considered as important biomarker in both models. Sphingolipids can regulate cell growth, differentiation, death and many important signal transduction pathways^41^. It was reported that phytosphingosine inhibited fibrosis by regulating the expression and activity of peroxisome proliferator-activated receptor γ (PPARγ)^42^. PPARγ has various biological functions, such as anti-inflammatory, anti-fibrotic, regulation of lipid metabolism by regulating expression of transforming growth factor β (TGF-β) and nuclear factor kappa B (NF-κB), inhibition of the proliferation of mesangial cells and reduction in the accumulation of extracellular matrix and type I collagen^43^. In addition, S1P plays an important role in the progression of lung fibrosis. Ikeda’s study found significant changes in the levels of S1P and its receptor in the liver tissues of patients with fibrosis^44^. Another report demonstrated that the role of S1P in fibrosis was correlated with the site of action and extracellular S1P promoted fibrotic processes in a S1P receptor-dependent manner, whereas intracellular S1P had an opposite effect^45^.

The biomarkers indicate abnormal sphingolipid metabolism in both models^34^. Compared with disease model, DS model includes additional TCM syndrome (qi-deficiency and blood-stasis, QDBS) on the base of abnormal serum biochemistry and pathological characteristics. Metabolic pathway analysis suggests DS model display more distinct abnormality in AA metabolism, linoleic acid metabolism and glycerophospholipid metabolism.

AA metabolic pathway includes AA together with its metabolites, LTE3 and 11b-PGF2α. AA mediates inflammation and the function of several organs and systems, either directly or upon its conversion into eicosanoids^46, 47^. The metabolites that are involved in the regulation of renal epithelial transport and vascular function, such as EETs and 20-HETE can regulate transport function in the proximal tubules, which is important to maintain renal function in patients with kidney disease by affecting Na^+^ **-**K^+^ **-**ATPase activity^48^. LTE3 is a by-product of the metabolism of LTC3, a cysteinyl leukotriene. Cysteinyl LTs (leukotrienes C4, D4 and E4) possess bronchoconstrictive and inflammatory effects in the lung via their action on specific leukotriene receptors. Montelukast, a potent cysteinylleukotriene1 (CysLT1) receptor antagonist, is therapeutically effective for the alleviation of further progression of lung fibrosis via the inhibition of α-smooth muscle actin (α-SMA). It was found that LT receptor blockers prevented necroinflammatory liver injury and fibrogenesis by modulating the hepatic expression of TGF-β, NF-κB, tissue inhibitor of metalloproteinase 1 and matrix metalloproteinases^49^. 11β-PGF2α is irreversibly produced from prostaglandin D2 via the enzyme prostaglandin-F synthase. Recent study demonstrated that PGD2 was probably involved in multiple aspects of inflammation through its dual receptor systems, DP and CRTH2^50^.

Linoleic acid pathway involves five metabolites of 15S-HETrE, docosahexaenoic acid (DHA), eicosapentaenoic acid (EPA), 9-HODE and 8,11,14-eicosatrienoyl ethanolamide. Among the five metabolites, 15S-HETrE and 9-HODE play a role in regulating PPARγ activity because 15S-HETrE can promote PPARγ expression^51^ and 9-HODE is an endogenous agonist of PPARγ^52^. 8,11,14-eicosatrienoyl ethanolamide is a DHA-derived N-acylethanolamine(NAE) metabolite, which modulates inflammation by reducing MCP-1 and NO production and expression^53^. DHA and EPA both belong to the long-chain ω-3 unsaturated fatty acid family, which maintains the normal physiological functions of cells and reduces blood cholesterol and triglycerides to improve blood microcirculation^31, 54, 55^. In addition, docosahexaenoic acid can suppress oxidative stress and fibrotic reaction in mice with non-alcohol fatty liver disease^56^. Moreover, EPA can regulate the ratio of urinary albumin/creatinine, as well as reduce the extracellular matrix accumulation of mesangial cells and renal tubulointerstitial fibrosis by adjusting monocyte chemoattractant protein-1 (MCP-1) levels^57^.

Lysophosphatidylcholines (LysoPCs) play a key role in process of glycerophospholipid metabolism, which have a pro-fibrotic effect because it can promote epithelial cell apoptosis, increase vascular permeability, promote fibroblast migration and anti-apoptosis, activate TGF-β activity and increase the secretion of the pro-fibrotic cytokines platelet-derived growth factor β and connective tissue growth factor in proximal tubule cells^58–62^. Moreover, LysoPCs can promote oxidative stress and enable cell structural and functional abnormalities that ultimately induce hemodynamic disorders by inhibiting Na^+^-K^+^-ATPase activity^35, 63, 64^.

Moreover, compared with disease model, some other particular biomarkers were also identifed in DS model. Phytosphingosine could regulate PPARγ expression and activity^42^, and its reduced level was closely related to the progress of fibrosis. tri-N-acetyl chitotriose, a component of oligosaccharides, could protect mucosal and maintain normal digestive action, and the reduced level of tri-N-acetyl chitotriose in model rats may be correlated with gastrointestinal dysfunction^65^. Estradiol is closely related to oxidative stress in patients with CKD as an anti-fibrosis component that inhibits the generation of reactive oxygen species and loss of anti-oxidant enzymes via the suppression of NADH/NADPH oxidase activity, attenuation of hydrogen peroxide induced TGF-β1 expression as well as proliferation and transformation of hepatic stellate cells^66, 67^.

The metabolomic study identified the similarities and differences of the two models. Common potential biomarkers related to abnormal sphingolipid metabolism were identified in disease model and DS model, which are associated with the formation of fibrotic cells or tissue and their cytokines, such as PPARγ, TGF-β and MCP-1. Furthermore, compared with the disease model, DS model shows its differences on AA metabolism, linoleic acid metabolism and glycerophospholipid metabolism, which were mainly involved in the maintenance of cell structure and function, regulation of cytokines related to fibrosis and other aspects and moderation of oxidative stress and inflammation. Accumulated evidences have demonstrated the presence of inflammation, oxidative stress, lipid metabolism disorders and other symptoms in patients with CKD. Therefore, DS model is in more coincidence with clinical settings.

## Conclusion

Disease model and DS model show no difference in clinical biochemistry, while metabolomics may highlight their difference. Abnormal sphingolipid metabolism is a common characteristic of both models, while AA metabolism, linoleic acid metabolism and glycerophospholipid metabolism are highlighted in DS model.

DS model not only involves the histopathological changes or abnormal biochemistry but can reveal potential biomarkers related to renal fibrosis, oxidative stress, inflammation and digestive disorders that are presented patients with CKD. This result is consistent with actual clinical situation. This consistence is beneficial for the translational medicine research and presents a greater advantage in the evaluation of disease and in the pharmacological study of drugs.
